# Starch coating with carbon dots: A promising coating to enhance the freeze–thaw stability of meatballs

**DOI:** 10.1016/j.fochx.2025.103169

**Published:** 2025-10-14

**Authors:** Linlin Zhao, Huinan Jiang, Zhengxuan Han, Wenqin Gu, Xiangren Meng

**Affiliations:** a College of Tourism and Culinary Science, Yangzhou University, Yangzhou 225127, Jiangsu, China; b Key Laboratory of Chinese Cuisine Intangible Cultural Heritage Technology Inheritance, Ministry of Culture and Tourism, Yangzhou 225127, China; c College of Food Science and Engineering, Yangzhou University, Yangzhou 225127, Jiangsu, China; d Chinese Cuisine Promotion and Research Base, Yangzhou 225127, Jiangsu, China

**Keywords:** Carbon dots, Starch, Meatballs, Freeze-thaw cycles, Water holding capacity

## Abstract

In this study, composite coatings were developed by incorporating carbon dots (CDs) to cross-linked starch (S). The effects of CDs on the coating structure and the quality of meatballs during freeze-thaw cycles were investigated. The results showed that after five freeze-thaw cycles, the sample containing 0.3 mg/mL CDs (S + 0.3CDs) exhibited significant structural and functional improvements compared to the S group. The short-range order of starch, represented by the R_1047/1022_, decreased to 1.15, accompanied by a 3.14 % reduction in crystallinity and a 30.52 % decrease in syneresis rate. These modifications contributed to enhanced meatball quality: The freeze-thaw loss, cooking loss, thiobarbituric acid reactive substances, and total bacterial count decreased by 4.24 %, 22.34 %, 29.68 %, and 19.25 %, respectively. Additionally, the S + 0.3CDs coating delayed lipid oxidation and microbial proliferation during storage. This study demonstrates that the addition of CDs optimally improves the freeze-thaw stability of the coating and the preservation effect of meatballs, providing a promising strategy for the development of high-performance coatings for frozen foods.

## Highlights

• CDs improved the freeze-thaw stability of starch coatings.
• CDs reduced starch crystallinity and short-range order, enhancing coating stability.
• S + CDs coating increased water holding capacity in meatballs, minimizing freeze-thaw loss.
• S + CDs coating delayed lipid oxidation and microbial growth of meatballs during freeze-thaw cycles.

## 1 Introduction

Frozen storage serves as the primary preservation method for meat products, yet temperature fluctuations during cold chain logistics inevitably undergo repeated freeze-thaw cycles (Zhang, Yu, et al., 2023), leading to multi-dimensional quality deterioration. Repeated freezing and thawing result in repeated ice crystal generation and melting. The ice crystal formation and melting dynamics will cause mechanical damage to muscle fibers (Song et al., 2017), induce cross-linking network breakage (M. Zhang, Li, Diao, Kong, & Xia, 2017), reduce water holding capacity (WHC) (H.-W. Kim, Kim, Seo, Setyabrata, & Kim, 2018), and accelerate microbial reproduction due to surface temperature variations (G.-D. Kim et al., 2013). Spoilage bacteria secrete proteases that degrade muscle proteins, accelerating deterioration in quality (J. Wang, Xie, & Mei, 2025). Concurrently, these processes induce protein oxidation and lipid oxidation, further exacerbating texture and flavor degradation. For instance, malondialdehyde disrupts the integrity of the phospholipid bilayer in cell membranes, accelerating moisture loss. In industrial production, the maintenance of frozen meat products**'** quality commonly relies on water retention agents, preservatives, and vacuum packaging technologies. Phosphate-based water retention agents can enhance WHC and reduce freeze-thaw losses (Kittiphattanabawon, Maqsood, Visessanguan, & Benjakul, 2023), but their application at elevated concentrations may affect natural flavor. Nitrites function effectively as color-fixing preservatives by inhibiting bacterial growth and maintaining vibrant meat color (Kia, Arief, Sumantri, & Budiman, 2015). However, their use may produce the potent carcinogen nitrosamines under acidic or high-temperature conditions. Vacuum packaging inhibits aerobic bacteria and lipid oxidation by isolating oxygen (Park, Hwang, Kang, & Kim, 2025), but vacuum compression may cause product deformation and proves ineffective against anaerobic microorganisms, such as lactic acid bacteria. Therefore, it is imperative to develop novel preservation techniques to improve frozen-storage quality and prolong shelf life in meat products.

Coating technology is widely applied in diverse fields such as food, medicine, packaging, etc. Forming a thin coating on the surface of objects, it enhances functional properties including barrier properties, mechanical strength, and antimicrobial efficacy (Abdel Aziz & Salama, 2022). Coating materials mainly include synthetic polymers (e.g., polyvinyl alcohol) and natural polymers (e.g., starch, protein, chitosan) (T. Wang et al., 2023). Starch coatings have become a research hotspot because of their degradability, food safety, and low cost. The film-forming mechanism of starch coating is based on the pasting and gelatinization, where hydrogen bonding and entanglement between molecular chains drive the formation of the coating layer (Thakur et al., 2019). Despite their advantages, starch coatings exhibit significant drawbacks: poor water resistance, insufficient mechanical strength, and suboptimal barrier performance (particularly against water vapor) (Jansson & Järnström, 2005). Starch molecules contain a lot of hydroxyl groups, rendering the coatings highly susceptible to water absorption, swelling, and even dissolution. Therefore, modifying starch is essential to enhance its physicochemical properties and functional attributes (including processing characteristics).

Cross-linked starch (CS), a type of modified starch, although it has enhanced the molecular network structure through chemical bonds and improved water resistance and mechanical strength, still suffers high brittleness and poor flexibility. The coating is prone to cracking and is unable to adapt to the contraction and deformation of meat products during the freezing process. Moreover, pure CS lacks antibacterial activity and is unable to inhibit the microbial proliferation during the thawing stage. Therefore, in this study, CS was used as the coating matrix and carbon dots (CDs) were introduced for further modification. CDs are a type of carbon-based nanomaterial with a size smaller than 10 nm, whose surface is enriched with organic functional groups such as carboxyl and hydroxyl groups, which attract much attention in recent years (Zhao, Zhang, Wang, & Devahastin, 2020). CDs possess excellent water dispersibility and bioconpatibility, while also featuring cost-effectiveness, stable photoluminescence properties, and significant antibacterial/antioxidant functions (Ezati et al., 2022). Compared to other existing nanomaterial modifications, CDs possess unique advantages of being non-toxic/low toxic, low-cost, and readily biodegradable. Riahi, Rhim, Bagheri, Pircheraghi, and Lotfali (2022) demonstrated that within a suitable concentration range, CDs exhibit no significant cytotoxicity toward L929 cells. It has been shown that the incorporation of CDs can enhance the functionality of the films, especially its antibacterial property. (Khan, Ezati, & Rhim, 2023). Xiao et al. (2019) prepared CDs/Chitosan coatings that could inhibit the growth of fungi and bacteria on mangoes, effectively reducing water loss and the rate of rotting. Fan, Zhang, Fan, and Jiang (2019) also prepared a CDs/chitosan coatings with excellent antibacterial effects, which could extend the shelf life of fresh cucumbers, show positive effects in maintaining vitamin C and reducing weight loss. Collectively, these studies demonstrate that CDs are widely used in the preparation of active packaging materials. Moreover, Wang, Yang, et al. (2020) has confirmed that CDs can inhibit ice growth and recrystallization. However, there are relatively few reports on the application of CDs in starch-based coatings and their use in improving the freeze-thaw stability of meat products.

This study constructed S + CDs composite coatings and systematically investigated changes in syneresis rate, microstructure, short-range ordered structure, crystallinity et al. of the coating gels after repeated freeze-thaw cycles. Through controlled freeze-thaw experiments of meatballs, the coating's protective effects on quality parameters, such as WHC, thiobarbituric acid value (TBARS), and total bacterial count (TBC), were quantitatively evaluated. The findings provide new insights for promoting the safe and standardized application of CDs in food engineering to address freeze-thaw challenges in cold chain logistics.

## 2 Materials and methods

### 2.1 Materials

Bananas, lean pork, pork back fat, and salt were purchased from the Farmers' Market in Yangzhou, China. Cross-linked starch (food grade) was bought from Henan Metro Industrial Co., Ltd. All other chemicals were purchased from Jiangsu Nanjing Bast Biological Co., Ltd.

### 2.2 Fabrication of CDs

CDs were hydrothermally synthesized following the method of Mehta, Jha, and Kailasa (2014). Briefly, 30 g of banana pulp was mixed with 100 mL of 30 % alcohol and homogenized for 3 min. Then transferred to a 50 mL hydrothermal reactor and heated in an oven at 180 °C for 6 h. Upon reaching ambient temperature, the reaction mixture was isolated by centrifugation (4000 rpm, 20 min), followed by membrane filtration of the supernatant (0.22 μm pore size). The resultant permeate underwent dialysis (MWCO: 500 Da) prior to lyophilization, yielding purified CDs powder. Finally, a 50 mg/mL solution of CDs was prepared in distilled water for subsequent experiments.

#### 2.2.1 Characterization of CDs

The morphological characteristics of the CDs were observed by transmission electron microscopy (TEM, JEOL, JEM-2100, Tokyo, Japan) at 200 kV. The X-ray diffraction (XRD) patterns of CDs were analyzed with an X-ray diffractometer (EDXS, Oxford Instruments, UK). The fourier transform infrared spectroscopy (FTIR) spectra of the CDs were obtained using an FTIR spectrometer (Thermo Fisher, Nicolet, iS10, Waltham, MA, USA). The UV/Vis absorption spectra of the CDs were determined using a UV/Vis spectrophotometer (PerkinElmer Lambda 35, Waltham, MA, USA). The fluorescence spectra of the CDs were recorded using a fluorescence spectrophotometer (Hitachi F-7000, Fukuoka, Japan).

#### 2.2.2 Measurement of antioxidant activity of CDs

The ABTS radical scavenging assay was performed following the method proposed by X. Li, Lin, Gao, Han, and Chen (2012). Briefly, ABTS solution (7.4 mmol/L) and potassium persulfate solution (2.6 mmol/L) were reacted in the dark for 12 h. The solution was diluted with 95 % ethanol to make A734 nm = 0.7 ± 0.02. 1.6 mL of the diluted ABTS solution was taken and mixed with 0.4 mL of CDs (0.0625, 0.125, 0.25, 0.5, and 1 mg/mL). After 30 min of reaction in the dark, a blank group was set up to eliminate the interference from CDs. The absorbance at 734 nm of ABTS solution (*A*_*0*_) and ABTS solution with CDs (*A*_*i*_) was recorded. The clearance rate was calculated as:

(1) $$\text{ABTS scavenging rate}\;\left(\%\right)=\frac{A_{0}-A_{i}}{A_{0}}\times 100$$

The DPPH radical scavenging assay was performed following the method proposed by Jamróz et al. (2019). 1 mL of freshly prepared ethanolic DPPH solution (0.1 mmol/L) was taken and mixed with 1 mL of CDs (0.0625, 0.125, 0.25, 0.5, and 1 mg/mL). After 30 min of reaction in the dark, a blank group was set up to eliminate the interference from CDs. The absorbance of DPPH solution (*A*_*0*_) and DPPH solution with CDs (*A*_*i*_) at 517 nm was recorded. The clearance rate was calculated as:

(2) $$\text{DPPH scavenging rate}\;\left(\%\right)=\frac{A_{0}-A_{i}}{A_{0}}\times 100$$

#### 2.2.3 Measurement of antimicrobial properties of CDs

Following the method of S. Zhang et al. (2020), the effect of CDs on the growth curves of *Escherichia coli* (*E. coli*) and *Staphylococcus aureus* (*S. aureus*) was tested, with minor modifications. In the experimental group, 100 μL of CDs solution (100, 50, 25, 12.5, 6.25, and 3.125 mg/mL) was mixed with 100 μL of bacterial inoculum (7 log10 CFU/mL) in a microplate, while the control group received 100 μL of sterile medium +100 μL of inoculum. The experiment also included 200 μL of medium alone as a sterile control. The cultures were incubated at 37 °C, and OD_600_ values were monitored at predetermined time points (0, 3, 6, 9, 12, 24, 36, and 48 h) to generate growth curves.

### 2.3 Preparation of S + CDs coating solution

Accurately weighed 70 g of CS, mixed it with 1 L of distilled water and stirred in an 85 °C water bath for 1 h. Then 200 mL, 199.6 mL, 199.2 mL, and 198.8 mL CS solution were taken and added with 0 mL, 0.4 mL, 0.8 mL, and 1.2 mL of a 50 mg/mL CDs solution, respectively. The samples were then continuously heated and stirred for 30 min before cooling to room temperature. The final concentrations of CDs were 0, 0.1, 0.2, and 0.3 mg/mL. The final coating samples were labeled as S, S + 0.1CDs, S + 0.2CDs, and S + 0.3CDs, respectively.

Freeze-thaw treatment: Each cycle consisted of a 22 h constant temperature curing stage at −20 °C, followed by a 2 h thawing process in a 30 °C water bath. All samples subjected to 0, 1, 3, and 5 freeze-thaw cycles. Freeze-thaw cycle tests were independently repeated three times.

### 2.4 Physicochemical and structural analysis of coatings during freeze-thaw cycles

#### 2.4.1 Viscosity

The viscosity was determined based on the method of Wójcik-Pastuszka, Iwaszkiewicz, and Musiał (2025) with minor modifications. The viscosity of the sample was determined by a rotational viscometer. The coating sample was placed in a beaker and kept at 30 °C water for 30 min. Select No. 4 rotor and set the speed at 6 r/min.

#### 2.4.2 Syneresis rate

The syneresis rate was determined based on the method of Mei, Huang, Bai, and Fu (2020) with minor modifications. The freeze-thaw samples were centrifuged at 4000 r/min for 10 min. The weight of the precipitates was accurately weighed. The syneresis rate was calculated as follows:

(3) $$\text{Syneresis rate}\;\left(\%\right)=\frac{m_{1}}{m_{2}}\times 100$$

• where, m_1_ is the weight of liquid separated from sample, g; m_2_ is the total weight of sample before centrifugation, g.

#### 2.4.3 FTIR analysis

The samples were subjected to freeze-drying after the freeze-thaw cycles and were subsequently ground into powder. The FTIR spectra of the samples were obtained by an FTIR spectrometer (Cary 610/670, Varian, USA) with a spectral range for scanning of 500–4000 cm^−1^.

#### 2.4.4 XRD test

The powder after freeze-drying was subjected to XRD (D8 Advance, Bruker AXS, Germany) testing and analysis. The scanning angle was set between 5 and 80° and the scanning speed was 6°/min.

#### 2.4.5 Scanning electron microscopy (SEM) analysis

The samples were subjected to freeze-drying after the freeze-thaw cycles. The cross-section of the dried samples were examined by SEM (GeminiSEM 300, Carl Zeiss GMBH, UK) with 200 × magnification.

### 2.5 Effect of S + CDs coatings on the freeze-thaw stability of meatballs

#### 2.5.1 Preparation of samples

Following the methodology of Shen, Zhang, and Yang (2024), with minor modifications. Lean pork and pork back fat (7:3, *w*/w) were minced using a meat grinder. The minced meat mixture was blended with 16 % (*v*/*w*) distilled water and 1.5 % salt (*w*/w) until a homogeneous emulsion formed. A portion of 50 g meat emulsion was shaped into meatballs. After boiling for 15 min, the meatballs were cooled to room temperature (25 ± 1 °C). The meatballs were respectively moistened in different coating solutions (S, S + 0.1CDs, S + 0.2CDs, and S + 0.3CDs) and then taken out, dried naturally to form films on the surface. The uncoated sample was used as the control group. There were three parallel samples in each group.

Freeze-thaw treatment: Following the methodology of Yun et al. (2025), with minor modifications. All samples were placed at −20 °C for 7 d. The samples were then thawed at 4 °C for 12 h, which was regarded as one freeze-thaw cycle. All samples subjected to 0, 1, 3, and 5 freeze-thaw cycles. Freeze-thaw cycle tests were independently repeated three times.

#### 2.5.2 Physicochemical analysis

##### 2.5.2.1 Thawing loss

Thawing loss was calculated based on the weight difference of meatballs before and after thawing, following the method of H.-W. Kim, Miller, Yan, Wang, and Kim (2017). The surface of the meatballs was wiped with filter paper to remove excess moisture, followed by immediate weighing. The calculation formula is as follows:

(4) $$\text{Thawing loss}\;\left(\%\right)=\frac{m_{1}-m_{2}}{m_{1}}\times 100$$

• where, m_1_ and m_2_ are the weights of meatballs before and after freeze-thaw, g.

##### 2.5.2.2 Cooking loss

The measurement of cooking loss was referred to the method of Zhu et al. (2022). The meatballs were placed in a steamer basket and steamed for 15 min after the water boiled. The weights of the meatballs before and after steaming were recorded. Prior to weighing, the surface of each meatball was gently wiped with filter paper to remove moisture. The formula is as follows:

(5) $$\text{Cooking loss}\;\left(\%\right)=\frac{m_{1}-m_{2}}{m_{1}}\times 100$$

• where, m_1_ and m_2_ are the weights of meatballs before and after steaming, g.

##### 2.5.2.3 WHC

Using the method of Wang, Zhou, et al. (2020), the thawed samples were centrifuged at 4 °C at 3000 ×*g* for 10 min, and the weights of the samples before and after centrifugation were recorded. The calculation formula is as follows:

(6) $$\mathrm{WHC}=\frac{m_{2}}{m_{1}}\times 100$$

• where, m_1_ and m_2_ are the weights of the samples before and after centrifugation, g.

##### 2.5.2.4 TBARS value

The method of Liu et al. (2022) was employed for the determination of TBARS. Briefly, 0.5 g of the sample, previously ground into small pieces, was mixed with 7.5 mL of trichloroacetic acid (TCA) solution. The mixture was homogenized under an ice bath, allowed to stand for 20 min, then filtered. Two milliliters of the filtrate was combined with 2 mL of 0.02 mol/L thiobarbituric acid (TBA) solution. Following the reaction at 100 °C for 40 min, the mixture was rapidly cooled to room temperature, and the absorbance at 532 nm was measured.

(7) $$\text{TBARS}/\left(\text{mgMDA}/\mathrm{kg}\right)=\frac{A_{532}-A_{600}}{155}\times \frac{1}{10}\times 72.6\times 100$$

• where, A_532_ is the absorbance at 532 nm; and A_600_ is the absorbance at 600 nm.

##### 2.5.2.5 pH value

The method of Wang, Zhang, and Ding (2020) was applied for the determination of pH. Briefly, 1 g of the meatball sample was weighed, ground, and subsequently mixed with 10 mL of distilled water. The mixture was homogenized and filtered, and the pH value of the resulting filtrate was measured.

##### 2.5.2.6 TBC

The TBC of the meatballs was determined according to the method of Yuan et al. (2022). The samples were mixed with sterile saline in a ratio of 1:9, thoroughly homogenized and diluted to different concentrations. The diluted solutions were poured into petri plates containing the culture medium and incubated at 37 °C for 48 h, then count the colonies using the plate count method. Four replicates were conducted for each sample. The results were expressed as log CFU/g.

### 2.6 Statistical analysis

All measurements were repeated three times, unless otherwise specified. The final results were expressed as mean ± standard deviation (SD). Statistical evaluation was conducted through one-way ANOVA with Duncan's multiple range test (α = 0.05) using IBM SPSS Statistics (v.26). Origin 2022 was used for mapping figures.

## 3 Results and discussion

### 3.1 Characterization and performance determination of CDs

#### 3.1.1 Characterization of CDs

**Fig. 1:**
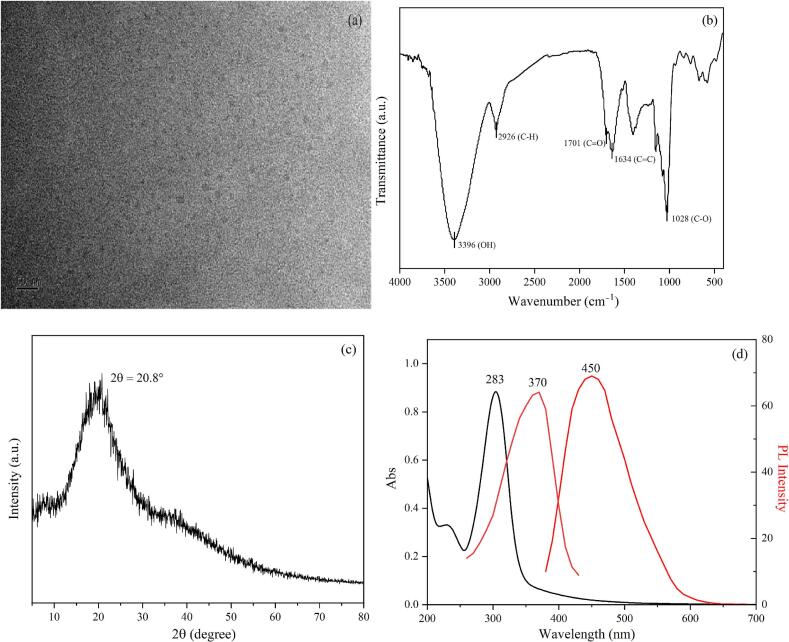
(a) TEM image, (b) FTIR spectra, (c) XRD patterns, (d) UV–Vis absorption spectra, PL excitation and emission spectra of CDs.

The results of the TEM are shown in Fig. 1a. It could be observed that the synthesized CDs exhibit a spherical structure, are uniformly dispersed, and have different particle sizes, similar to the CDs morphology reported by Riahi et al. (2022). Infrared spectroscopy was used to explore the structural characteristics of CDs, and the results are shown in Fig. 1b. The absorption peak at 3396 cm^−1^ reflected the stretching vibration of O—H, the absorption peak at 2926 cm^−1^ reflected the stretching vibration of C—H, the absorption peak at 1701 cm^−1^ and 1031 cm^−1^ represented the stretching vibration of C=H and C—O. It is indicated that there are a large number of hydroxyl and carboxyl functional groups on the surface of CDs, which makes them have good hydrophilicity. The XRD pattern is shown in Fig. 1c. It can be seen that a relatively broad diffraction peak appears at a 2θ angle of approximately 20.8°, which is related to the lattice spacing of graphite carbon. This indicated that the prepared CDs possess a structure similar to graphite (Zhang, He, et al., 2023). The analysis results of ultraviolet-visible spectroscopy are shown in Fig. 1d. The synthesized CDs exhibit a maximum ultraviolet absorption peak at 283 nm. This is believed to be caused by the π-π* electron transition on the C=C bond and the n-π* transition on the C=O bond contained in the CDs (Pudza et al., 2019), which indicates that the CDs structure has an sp^2^ carbon structure. Fig. 1d shows the fluorescence spectral analysis of CDs. The maximum excitation wavelength of the CDs is located at 370 nm, and the maximum emission wavelength is located at 450 nm.

#### 3.1.2 Antioxidant and antibacterial activity of CDs

**Fig. 2:**
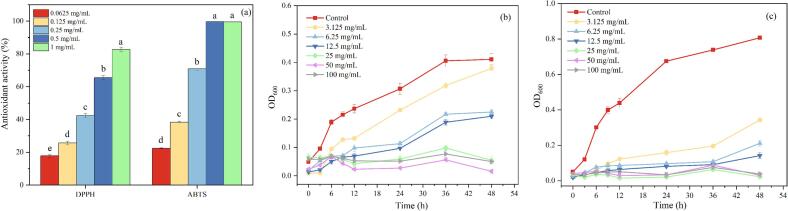
(a) DPPH and ABTS radical scavenging activities of CDs, and the growth curve of (b) *E. coli* and (c) *S. aureus* treated with different concentrations of CDs. Note: Different groups with different lowercase letter mean significantly different (*P* < 0.05).

Fig. 2a demonstrates the free radical scavenging activities of CDs. As the concentration of CDs increased, their scavenging rate against ABTS radicals rose from 22.5 % to 99.5 %, whereas that against DPPH radicals increased from 17.9 % to 82.7 %. This enhanced scavenging activity could be attributed to the surface functional groups of CDs. These groups facilitated the transfer of hydrogen atoms (H·) to the radicals, resulting in the formation of stable reduced forms (ABTS-H and DPPH-H) (Riahi et al., 2022). Consequently, a higher scavenging efficiency for ABTS radicals than for DPPH radicals was exhibited in CDs. This difference likely stemmed from the superior dispersion and stability of hydrophilic CDs in the aqueous ABTS assay system, which increased the exposure of reaction sites and enhanced interactions with the hydrophilic ABTS radicals.

The dose-dependent inhibitory effect was demonstrated through monitoring the bacterial growth curve (OD_600_, Fig. 2b-c). Increasing concentrations of CDs (0–100 mg/mL) progressively suppressed proliferation in both strains, confirming a concentration-response relationship. CDs possess unique physicochemical properties that confer antibacterial activity. The functional groups on their surfaces can interact with the lipid and protein components of bacterial cell membranes, disrupting membrane integrity and inducing structural instability. At higher concentrations, an increased number of CDs can bind more extensively and disrupt the membrane structure, enhancing its permeability and ultimately causing cell death, thereby strengthening the inhibitory effect. The inhibitory effect on *S. aureus* is significantly stronger than that on *E. coli*. This selectivity is related to the structural differences of the bacterial cell wall. Because Gram-negative bacteria have an additional outer membrane, it hinders the penetration of antibacterial agents.

### 3.2 Effects of CDs on the structure and properties of coatings during freeze-thaw cycles

#### 3.2.1 Viscosity

**Fig. 3:**
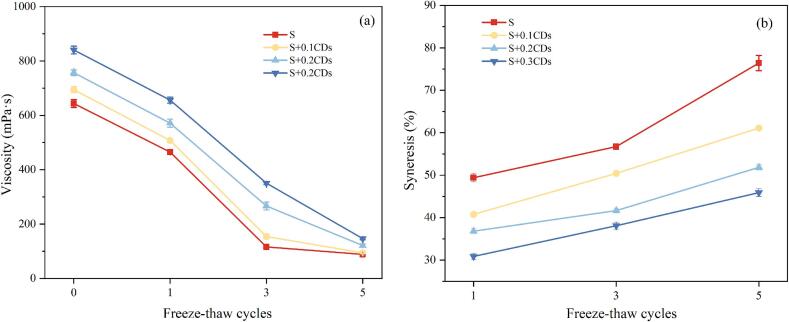
(a) Viscosity and (b) syneresis rate of the various coatings under different numbers of freeze-thaw cycles.

As shown in Fig. 3a, the viscosity of all experimental groups decreased progressively with increasing freeze-thaw cycles. This is because ice crystal formation during freeze-thaw process exerts mechanical shear forces on starch long-chain molecules, leading to chain cleavage and a reduction in molecular weight (L. Wang, Yin, Wu, Sun, & Xie, 2008). The decline in molecular weight directly diminished the hydration capacity and network strength of starch, thereby reducing viscosity. Importantly, higher CDs concentrations significantly increased the viscosity of the coating solution (*P* < 0.05). This enhancement resulted from the abundant functional groups (e.g., –OH, –COOH) on the CDs surfaces, which formed hydrogen bonds with starch molecules. The increased CDs concentration provided more cross-linking sites, while simultaneously inhibiting mechanical shear damage from ice crystals to starch chains. Consequently, CDs incorporation better preserved the integrity of the starch network structure and optimized viscosity stability (Lu, Guo, Fan, Wang, & Yan, 2023). The elevated viscosity improved food stability by reducing deformation or rupture risks during storage. Additionally, the highly viscous coating effectively retained moisture within the food matrix, prevented dehydration during drying or storage, and thereby maintained sensory texture and quality.

#### 3.2.2 Freeze–thaw stability

WHC reflects the ability of starch-based systems to retain water under specific conditions, correlating with the number of hydroxyl groups in the molecular structure. A lower syneresis rate indicates stronger WHC in starch gels. As shown in Fig. 3b, all experimental groups exhibited progressively higher syneresis rates after repeated freeze-thaw, indicating weakened freeze-thaw stability. During freezing, water molecules within the starch gel formed ice crystals; subsequent thawing converted these crystals back to water, which was expelled from the gel network. Repeated freeze-thaw cycles promoted progressive syneresis, inducing a porous, sponge-like structure with reduced WHC (Chen et al., 2023). Consistent with Guo, Wang, Liu, and Wang (2020), ice crystal formation during freeze-thaw cycles disrupts the gel structure, enlarging the voids inside the gel, and reducing accessible hydroxyl binding sites, thereby increasing water expulsion and decreasing WHC. Notably, higher CDs concentrations significantly decreased syneresis rates (*P* < 0.05), demonstrating concentration-dependent enhancement‌ of WHC. After five cycles, S + 0.2CDs and S + 0.3CDs samples maintained significantly lower syneresis rates than other groups (*P* < 0.05), confirming superior WHC. This improvement was attributed to CDs stabilizing the gel's microscopic network via hydrogen bonding with starch chains, inhibiting water expulsion, and preserving structural integrity. Consequently, CDs incorporation reduced water loss in starch composites and enhanced freeze-thaw stability.

#### 3.2.3 Short-range ordered structure

**Fig. 4:**
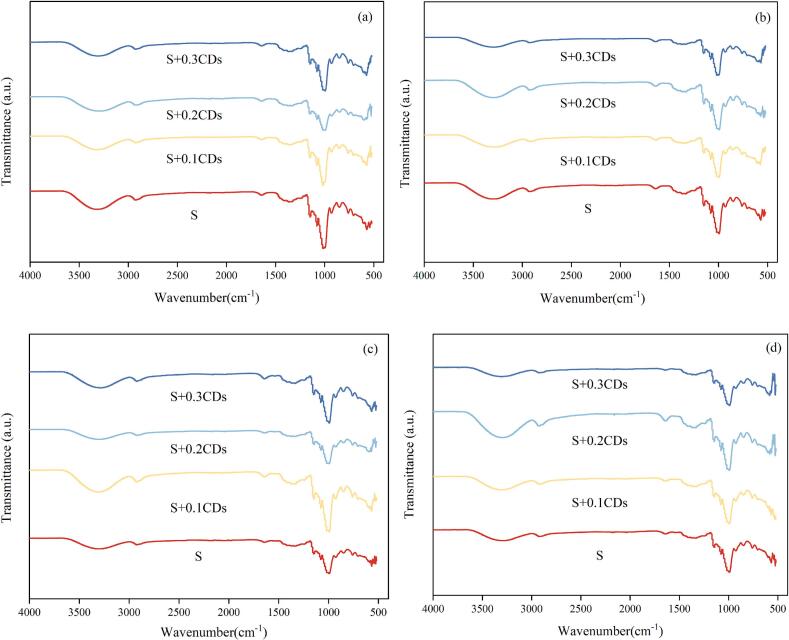
FTIR spectra of various coatings under (a) 0, (b) 1, (c) 3, and (d) 5 freeze-thaw cycles.

The crystalline structural changes of starch gels are related to the short-range ordered molecular structure. FTIR spectra of all samples post freeze-thaw cycles (Fig. 4a-d) exhibited consistent peaks positions and profiles across 4000–500 cm^−1^, with no emergent/disappearing peaks or no new functional groups. All starch gels had a broad and strong characteristic peak in the range of 3500–3200 cm^−1^ corresponding to stretching vibrations and hydrogen bond formation (Jia et al., 2022). Starch gels in the region of 1200–800 cm^−1^ have absorption peaks near 1047 cm^−1^ and 1022 cm^−1^, represented the ordered and disordered structures of starch, respectively. The intensity ratio of 1047 cm^−1^ to 1022 cm^−1^ quantitatively reflects the orderliness of starch gel, where higher ratios denoted higher orderliness (Q.-Z. Li, Chang, He, Chen, & Zhou, 2019). The intensity ratio of 995 cm^−1^ to 1022 cm^−1^ quantitatively reflects the double helix structure of the starch gel, with a higher ratio indicating a greater content of the double helix structure.

**Fig. 5:**
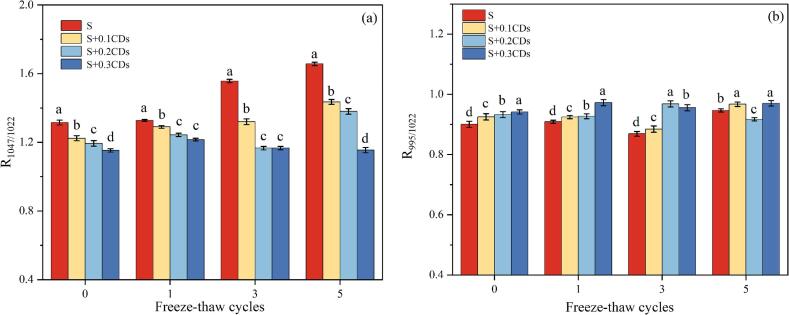
(a) R_1047/1022_ and (b) R_995/1022_ values of FTIR spectra of various coatings under different numbers of freeze-thaw cycles. Note: Different groups with different lowercase letter mean significantly different (*P* < 0.05).

As shown in Fig. 5, with the increase in the number of freeze-thaw cycles, the values of R_1047/1022_ in all groups significantly increased (*P* < 0.05). This trend indicated that freeze-thaw cycles facilitate the formation of short-range ordered molecular structures. These structural changes further demonstrated a progressive increase in starch retrogradation (Y. Zhang et al., 2014). After five freeze-thaw cycles, the R_1047/1022_ value of pure starch gels (Group S) increased from 1.31 to 1.65, indicating an improvement orderliness. In contrast, the R_1047/1022_ values of S + 0.1CDs, S + 0.2CDs, and S + 0.3CDs groups were 1.43, 1.37, and 1.15. All were significantly lower than those of group S (*P* < 0.05). These results demonstrated that CDs effectively inhibited the formation of ordered starch structures during freeze-thaw, thereby delaying retrogradation. Notably, the inhibitory effect intensified with higher CDs concentrations. The R_995/1022_ of the starch gel containing CDs was higher than that of the pure starch gel, indicating that the hydrogen bonds formed between starch molecules and CDs increased, inhibiting the rearrangement of starch chains and promoting the formation of double helix structures.

#### 3.2.4 Relative crystallinity

**Fig. 6:**
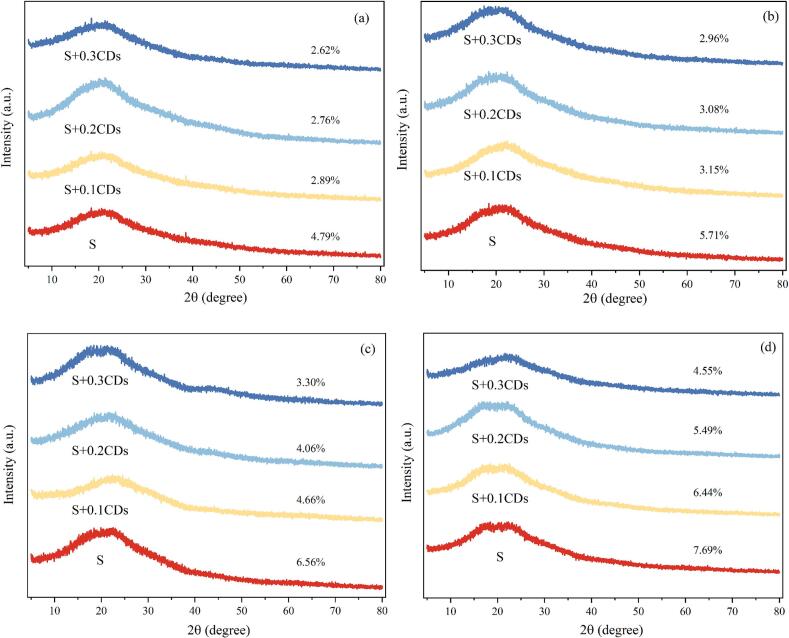
XRD patterns of various coatings under (a) 0, (b) 1, (c) 3, and (d) 5 freeze-thaw cycles.

The XRD patterns of coatings subjected to different freeze-thaw cycles are shown in Fig. 6. At 2θ = 20°, all samples showed broad diffraction peaks, indicating that the addition of CDs dose not affect the crystal structure of starch gel. During the freezing process of starch gel, the starch within the gel forms enriched regions, resulting in more compact double-helix structure. This lead to an increase in relative crystallinity, which in turn reflects a higher degree of starch retrogradation (Kuang et al., 2022). As the freeze-thaw cycles increased, the crystallinity of all gel samples exhibited a progressive enhancement. However, the relative crystallinity of starch gels containing CDs consistently remained lower than that of pure starch gels. The relative crystallinity of S group increased from 4.79 % to 11.78 % after five freeze-thaw cycles, while the values of S + 0.1CDs, S + 0.2CDs, and S + 0.3CDs group were 6.44 %, 5.49 %, and 4.55 %, respectively. These results demonstrate that the incorporation of CDs inhibited the recrystallization of starch chains and delayed the the retrogradation of starch.

#### 3.2.5 Microstructure

**Fig. 7:**
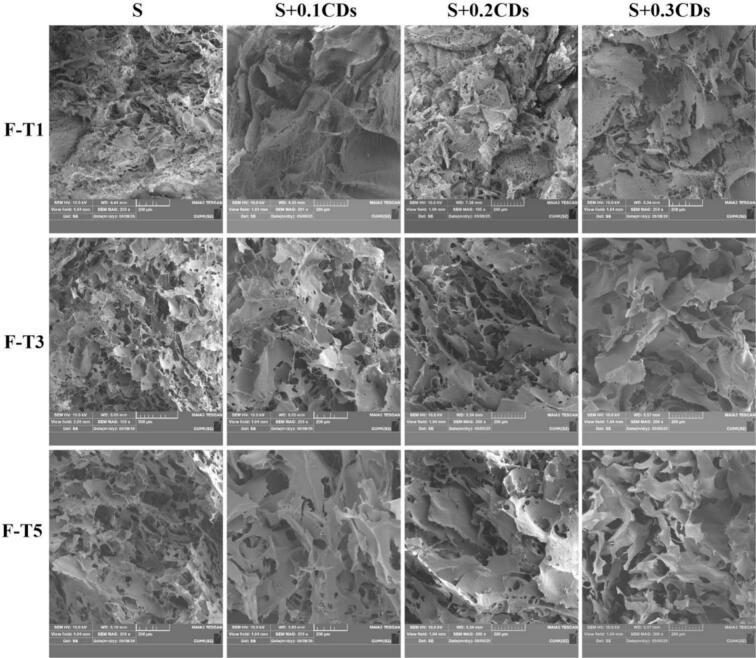
SEM images of various coatings under 1, 3, and 5 freeze-thaw cycles.

Fig. 7 presents the SEM images of pure starch gel and CDs-incorporated starch gel after freeze-thaw cycles (1, 3, and 5 cycles). Pure starch gel forms a network structure after freeze-thaw cycles. This structure is the result of the combined effect of the formation of ice crystals and the orderly rearrangement of starch (Y. Fu et al., 2023). Compared to the pure starch gel, the starch gels with CDs exhibited smaller pores after one freeze-thaw cycle, particularly in the S + 0.3CDs gel with a CDs concentration of 0.3 mg/mL, which showed the fewest pores. After 3 and 5 freeze-thaw cycles, the pore size in S + 0.1CDs, S + 0.2CDs, and S + 0.3CDs groups increased, but the number of pores remained lower than that in the S group. Furthermore, the incorporation of CDs enhanced the continuity and integrity of the gels. Combined with the results of syneresis rate, it can be concluded that the addition of CDs inhibited ice crystal formation, reduced the overflow of liquid water converted from ice crystals, mitigated syneresis, and reflected the excellent WHC of the S + CDs gels.

### 3.3 Effect of S + CDs coating on the freeze-thaw stability of meatballs

#### 3.3.1 Thawing loss

**Fig. 8:**
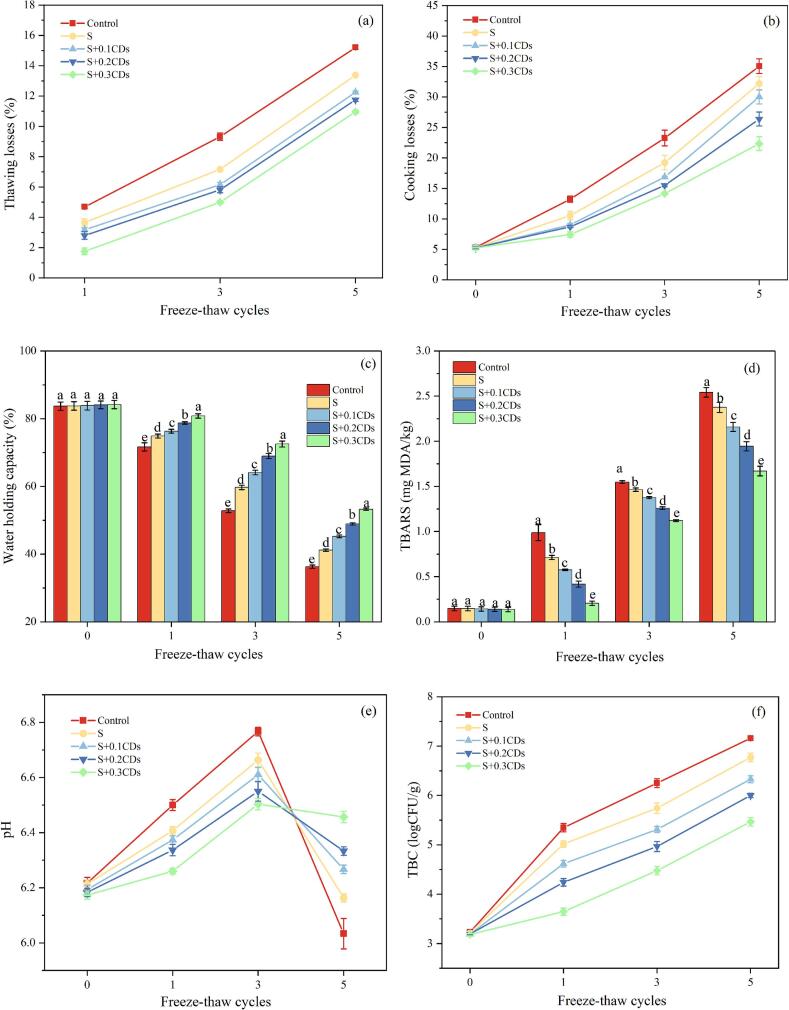
Effect of different coatings on (a) thawing loss, (b) cooking loss, (c) WHC, (d) TBARS, (e) pH and (f) TBC of meatballs under different numbers of freeze-thaw cycles. Note: Different groups with different lowercase letter mean significantly different (*P* < 0.05).

Thawing loss served as a critical indicator of meatball quality deterioration (Xia, Kong, Liu, & Liu, 2009). Repeated freeze-thaw cycles damaged the internal microstructure of meatballs, compromising their water-retention capacity. As shown in Fig. 8a, the thawing loss rate increased significantly (*P* < 0.05) with additional freeze-thaw treatments in all samples. However, the S + CDs coating treatment significantly reduced thawing loss (*P* < 0.05), and this inhibitory effect exhibiting a concentration-dependent response within the tested range. Higher CDs concentrations demonstrated more pronounced suppression of thawing loss. This phenomenon may be attributed to the coating's ability to inhibit intracellular water migration during the freezing phase, while reducing water exudation caused by cell rupture during thawing, thereby decreasing thawing loss. This is similar to the study by Cardoso et al. (2016), which demonstrated that polysaccharide-based edible coatings can form protective layers on food surfaces, effectively minimizing moisture loss. Further analysis suggested that CDs cross-linked with starch via hydrogen bonding, while their nano-scale size (< 10 nm) filled micropores and cracks within the starch film. SEM results also confirm that after multiple freeze-thaw cycles, the ice crystals formed in S + CDs coatings are smaller and more densely structured. Consequently, this generated a denser coating structure that effectively blocked moisture migration, suppressed ice crystal growth, and enhanced thawing loss control. In addition, the S + CDs coatings demonstrate a lower syneresis rate, which also reduce the moisture loss of the samples to a certain extent.

#### 3.3.2 Cooking loss

Fig. 8b demonstrates the effect of different coatings on the cooking loss of meatballs after different freeze-thaw cycles. Cooking loss serves as a critical indicator of water retention capacity in meat products. As the number of freeze-thaw cycles increased, all samples showed a notable increase in cooking loss (*P* < 0.05). The starch coating formed a dense gel layer on the surface of the meatballs, which effectively blocked the loss of water and thus reduced the cooking loss, similar to the findings of Azadi, Rafieian, Sami, and Rezaei (2023). As the concentration of CDs increased, the structure of the S + CDs coating became more compact, which effectively reduced cooking loss. The three-dimensional network structure formed through hydrogen bonding between CDs and starch molecules, along with their nano-size, filled the micropores or cracks in the starch coating, creating a denser structure that significantly reduced the water vapor permeability and enhanced mechanical strength, thus blocking water migration more effectively.

#### 3.3.3 WHC

A higher WHC value signifies less structural damage caused by ice crystal formation during repeated freeze-thaw cycles. As shown in Fig. 8c, the WHC of samples exhibited a significant decline (*P* < 0.05) with increasing freeze-thaw cycles, consistent with the results of Jiang, Nakazawa, Hu, Osako, and Okazaki (2019). Research indicates that during freeze-thaw cycles, the melting and recrystallization of ice crystals cause mechanical damage to muscle tissue. The formed ice crystals pierce the sarcolemma of myofibrillar cells, leading to the collapse of the myosin (such as actin and myosin) network and the subsequent leakage of intracellular water. Under identical freeze-thaw cycle conditions, starch coating group held higher WHC compared to the control group. Moreover, the incorporation of CDs further enhances the WHC of starch coatings. The polar functional groups enriched on the surface of CDs can captured free water molecules through hydrogen bonding, reducing the content of freezable water during freezing, and promoting the formation of smaller, more uniformly distributed ice crystals. This mechanism minimizes mechanical damage to muscle tissues, thus mitigates the decline in WHC during freezing and thawing. Similar to the findings of Ding et al. (2024), the abundant hydroxyl groups on the surfaces of P-CDs and starch/polyvinyl alcohol films facilitate stable bonding through hydrogen bonding, greatly enhancing the hydrophilicity of the films. Hydrogen bond networks immobilize water molecules, inhibiting the crystallization of free water during freezing. Hydrophilic surfaces reduce interfacial tension between ice crystals and substrates, minimizing mechanical damage from ice crystal penetration and reducing moisture loss.

#### 3.3.4 TBARS

The TBARS values of the samples increased significantly (*P* < 0.05) with the increase of freeze-thaw cycles (Fig. 8d), which can be attributed to the high-fat content of the meatball samples, which is susceptible to oxidation. With deeper oxidation, hydroperoxides are formed from unsaturated fatty acids and subsequently decomposed into MDA and other carbonyl compounds that contribute to undesirable flavors (Alirezalu et al., 2019). After five freeze-thaw cycles, the TBARS value of the control group increased to 2.54 mg MDA/kg. The TBARS values of starch-coated meatballs (S group) were lower than that of the control group, which may be attributed to the starch coating forming an oxygen barrier on the surface of the meatballs, thereby better inhibiting oxidative deterioration. Similar to the findings of Hu, Yong, Zong, Jin, and Liu (2022), chitosan coating showed excellent inhibitiory effects on lipid oxidation. The addition of CDs further enhanced the coating's efficacy in reducing the TBARS value of meatballs, with the S + 0.3CDs coating showing the best effect. CDs had excellent antioxidant properties and conferred antioxidant properties to the starch composite coating, effectively scavenged free radicals, thus reducing the TBARS value of meatballs. Consistent with the research conducted by Koshy et al. (2021), who prepared pH-sensitive biodegradable starch films containing CDs and anthocyanins, CDs enhanced the mechanical properties and water resistance of the starch films while imparting antioxidant properties.

#### 3.3.5 pH value

The oxidation of proteins and lipids usually occurs during the storage of meat products. The byproducts of these oxidative reactions can decrease the pH value, leading to product deterioration. A series of internal changes may occur within the meatballs, such as glycogenolysis, muscle components degradation, and proteolysis, which can adversely affect the quality of the meatballs (M. Zhang, Haili, Chen, Xia, & Kong, 2018). As shown in Fig. 8e, the pH value of the samples initially increased and then decreased. Repeated freeze-thaw will lead to the rupture of muscle cells, releasing proteases that break down proteins into alkaline substances (e.g., ammonia), potentially leading to a slight increase in pH. If temperature control is inadequate during the freeze-thaw process, microbial activities may produce acidic metabolites (e.g., lactic acid), resulting in an initial rise followed by a decline in pH. The three S + CDs coating groups exhibited superior antioxidant activity and antimicrobial properties due to the incorporation of CDs. As the concentration of CDs increased, the effect of alleviating pH changes in meatballs became more prominent. This phenomenon can be attributed to the effective inhibition of bacterial proliferation by CDs, which also delays the decomposition of proteins and amino acids in the meatballs. Similar to the findings of H. Zhang, Zheng, and Li (2022), ascorbic acid, ε-polylysine, and chitosan-based coating treatments slowed down the breakdown of proteins and amino acids in pork and suppressing the decrease in pH.

#### 3.3.6 TBC

Fig. 8f illustrates the effect of different coating treatments on the TBC of meatballs during repeated freeze-thaw cycles. Meatballs are susceptible to microbial contamination during freeze-thaw cycles, and the temperature during thawing is conducive to microbial proliferation. The TBC of meatballs significantly increased (*P* < 0.05) with progressive freeze-thaw cycles. Starch coating treatment notably suppressed TBC elevation, but its efficacy was markedly inferior to three S + CDs composite coatings. Furthermore, The S + CDs coatings demonstrated concentration-dependent inhibition, with higher CDs concentrations (0.1–0.3 mg/mL) yielding lower TBC values at equivalent cycles. This finding aligns with the results of B. Fu et al. (2022), where CDs-reinforced gelatin/chitosan films showed superior TBC control in fish compared to unmodified films, suggesting a generalized antimicrobial mechanism of CDs-enhanced coatings in natural polymer matrices.

## 4 Conclusions

In this study, CDs-modified starch coating was prepared. The incorporation of CDs improved the freeze-thaw stability of the starch coating and contributed to the improvement of frozen storage stability of meatballs. XRD and FTIR analyses revealed that CDs effectively inhibited the recrystallization of starch chain as well as the formation of short-range ordered structures, thereby retarding the retrogradation process. The SEM analysis ‌demonstrated that CDs suppressed ice crystal formation, resulting in a more compact starch gel network structure. The freeze-thaw experiments on meatballs further indicated that the S + CDs coating reduced the thawing loss and cooking loss, improved the WHC, and delayed the increase in TBC and TBARS values of the meatballs. The composite coating with 0.3 mg/mL CDs exhibited optimal performance. These findings suggest that the S + CDs coating is beneficial for enhancing the freeze-thaw stability of meat products and holds promising application prospect in improving the quality of frozen products.

## CRediT authorship contribution statement

**Linlin Zhao:** Writing – review & editing, Supervision, Resources, Project administration, Funding acquisition, Conceptualization. **Huinan Jiang:** Writing – original draft, Validation, Investigation, Formal analysis, Data curation. **Zhengxuan Han:** Methodology, Investigation, Formal analysis, Data curation. **Wenqin Gu:** Validation, Investigation. **Xiangren Meng:** Writing – review & editing, Supervision, Resources.

## Declaration of competing interest

The authors declare that they have no known competing financial interests or personal relationships that could have appeared to influence the work reported in this paper.

## Data Availability

Data will be made available on request.
